# Chinese Medicine Shenfu Injection for Heart Failure: A Systematic Review and Meta-Analysis

**DOI:** 10.1155/2012/713149

**Published:** 2012-04-24

**Authors:** Song Wen-Ting, Cheng Fa-Feng, Xu Li, Lin Cheng-Ren, Liu Jian-Xun

**Affiliations:** ^1^Xiyuan Hospital, China Academy of Chinese Medicine Sciences, Beijing 100091, China; ^2^School of Basic Medicine, Beijing University of Chinese Medicine, Beijing 100029, China

## Abstract

*Objective*. Heart failure (HF) is a global public health problem. Early literature studies manifested that Shenfu injection (SFI) is one of the most commonly used traditional Chinese patent medicine for HF in China. This article intended to systematically evaluate the efficacy and safety of SFI for HF. *Methods*. An extensive search was performed within 6 English and Chinese electronic database up to November 2011. Ninety-nine randomized controlled trails (RCTs) were collected, irrespective of languages. Two authors extracted data and assessed the trial quality independently. RevMan 5.0.2 was used for data analysis. *Results*. Compared with routine treatment and/or device support, SFI combined with routine treatment and/or device support showed better effect on clinical effect rate, mortality, heart rate, NT-proBNP and 6-minute walk distance. Results in ultrasonic cardiography also showed that SFI combined with routine treatment improved heart function of HF patients. There were no significant difference in blood pressure between SFI and routine treatment groups. Adverse events were reported in thirteen trails with thirteen specific symptoms, while no serious adverse effect was reported. *Conclusion*. SFI appear to be effective for treating HF. However, further rigorously designed RCTs are warranted because of insufficient methodological rigor in the majority of included trials.

## 1. Introduction

Heart failure (HF) is a leading cause of death, hospitalization, and rehospitalization worldwide. Despite advances in the treatment of HF, including use of drugs, devices, and heart transplantation, the condition remains associated with substantial morbidity and mortality [1].

International cooperation research program on cardiovascular disease in Asia showed that, on a total of 15,518 Chinese adults (35–74 years old) survey, the prevalence of HF was 0.9%, 0.7% for the males, and 1.0% for the females [2]. In the United States, HF incidence approaches 10 per 1,000 of the population over 65 years of age [3]. A report from the European Society of Cardiology (ESC) indicated at least 10 million patients with HF in these representing countries with a population of over 900 million. Half of the HF patients will die within 4 years, and more than half of those with severe HF will die within 1 year [4]. 

At present, the conventional therapeutic approaches in HF management include angiotensin-converting enzyme (ACE) inhibitors, *β*-blockers, and diuretics. Although several of them have led to an important effectiveness, HF remains the leading cardiovascular disease with an increasing hospitalization burden and an ongoing drain on health care expenditure [5]. Therefore, it remains necessary to search alternative and complementary treatment, in which Traditional Chinese Medicine takes a good proportion [6].

**Figure 1:**
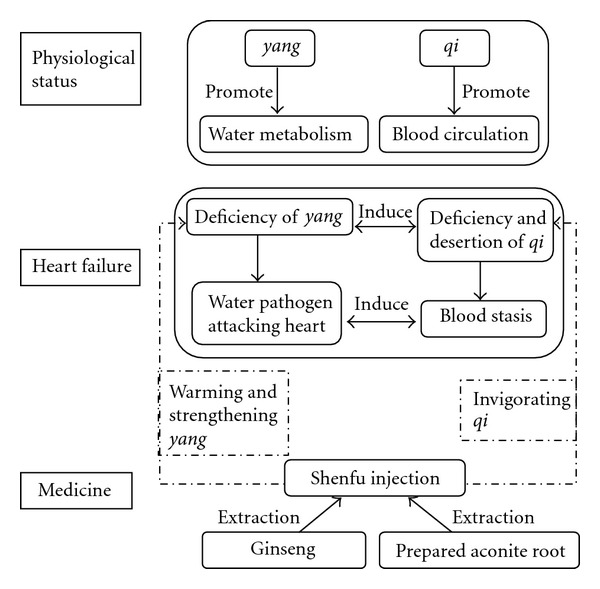
TCM theory on heart failure and Shenfu injection.

In TCM theory, pathogenesis of HF is related to deficiency of heart *yang* and heart *qi* and stasis of *blood* and excessive *water (fluid)*, as well as interaction within these pathological factors. Under physiological conditions, *yang* can promote *water* metabolism, while *qi* can accelerate *blood* circulation, so *yang* and *qi* are the vital elements for human body to maintain life activity. TCM theory holds that patients suffered from HF are in deficiency of heart *yang* and *qi* for a long course, which directly leads to excessive *fluid* retention and *blood* stasis (Figure 1).

Two Chinese herbal medicines, namely, Radix *Ginseng* (ginseng) and Radix *Aconiti Lateralis Preparata* (prepared aconite root), are used in treating HF over 2000 years. Ginseng invigorates *qi*, while prepared aconite root can warm and strengthen yang and lead to diuresis. Long-term clinical practice has proved that compatibility of ginseng and prepared aconite root can effectively ameliorate patients' symptom of HF and improve quality of life (Figure 1).

Shenfu injection (SFI) has been used in treating cardiac diseases for a long time in China [7]. The main active components of SFI are extraction of traditional Chinese herbs, namely, ginsenosides and higenamine. Modern pharmacological research shows that ginsenosides can improve ischemic myocardium metabolism, scavenge free radicals, protect myocardial ultrastructure, and reduce Ca^2+^ overload, and higenamine can enhance heart contractility, improve coronary circulation, and decrease the effect of acute myocardial ischemia [8].

Currently, SFI used alone or integrated with routine treatments has been widely accepted as an effective method for the treatment of HF in China. Many clinical studies reported the effectiveness ranging from case reports and case series to controlled observational studies and randomized clinical trials, but the evidence for its effect is not clear. This paper aims to evaluate the beneficial and harmful effects of SFI for treatment of HF in randomized controlled trials.

## 2. Methods

### 2.1. Database and Search Strategies

A systematic search was conducted in 5 databases including PubMed (1980–2011), China National Knowledge Infrastructure (1994–2010), VIP Database for Chinese Technical Periodicals (1979–2010), Chinese Biomedical Literature Database (1995–2011), and Cochrane Library (Issue 10, 2011), with the following terms: (Shenfu injection or Shenfu or Shen-fu) AND (heart failure or cardiac dysfunction or cardiac inadequacy or cardiac failure or congestive heart failure). All of those searches ended before November 2011. And the bibliographies of included trials were searched for thorough references, irrespective of languages.

### 2.2. Inclusion Criteria

All the randomized controlled trails (RCTs) of SFI compared with routine or conventional treatment (control group) in adult patients with HF were included. RCTs combined SFI with conventional treatment and/or invasive respiratory support (SFI group) compared with conventional treatments and/or invasive respiratory support (control group) were included. Both acute heart failure and chronic heart failure were included. Outcome measures include clinical effect rate, death and adverse events, ultrasonic cardiography, heart rate and blood pressure, and quality of life.

### 2.3. Data Extraction and Quality Assessment

**Figure 2:**
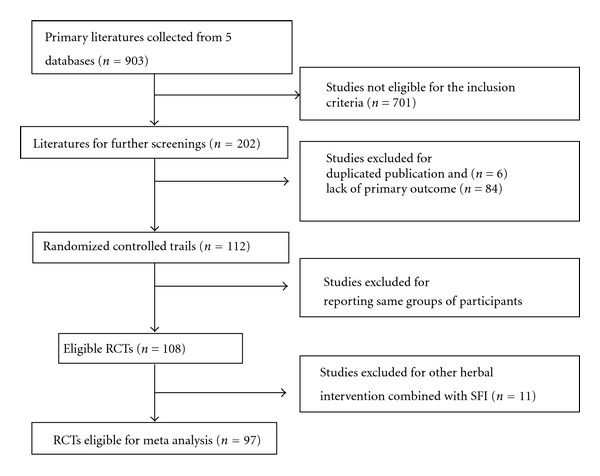
Diagram of the study selection flow.

Two authors (S. Wen-Ting and C. Fa-Feng) extracted the data from the included trials independently, based on the inclusion criteria outlined above. Nonrandomized evaluations, pharmacokinetic studies, animal/laboratory studies, and general reviews were excluded, and duplicated publications reporting the same groups of patients were also excluded (Figure 2).

Extracted data was entered into an electronic database by two authors, S. Wen-Ting and C. Fa-Feng independently. The methodological quality of RCTs was assessed by using criteria from the Cochrane Handbook for Systematic Reviews of Interventions, Version 5.0.1. The quality of trials was categorized into low risk of bias, unclear risk of bias, or high risk of bias according to the risk for each important outcome within included trials, including adequacy of generation of the allocation sequence, allocation concealment, blinding, whether there were incomplete outcome data or selective outcome, or other sources of bias.

### 2.4. Data Synthesis

 The statistical package (RevMan 5.0.2), which is provided by The Cochrane Collaboration, was used to analyze collected data. Dichotomous data was presented as risk ratio (RR), with 95% confidence intervals (CIs). Continuous outcomes were presented as mean difference (MD), with 95% CI. Analyses were performed by intention-to-treat where possible. Heterogeneity between trials results was tested, and heterogeneity was presented as significant when *I* ^2^ is over 50% or *P* < 0.1. Random effect model was used for the meta-analysis if there was significant heterogeneity, and fixed effect model was used when the heterogeneity was not significant [21]. Publication bias was explored via a funnel-plot analysis. 

## 3. Result

### 3.1. Search Flow

According to the search strategy, we screened out 903 potentially relevant studies for further identification (Figure 2). By reading titles and abstracts, we excluded 701 studies that were obviously ineligible, including review articles, case reports, animal/experimental studies, and nonrandomized trials. 202 studies with full text papers were retrieved. After the full text reading, 6 studies were excluded because of duplicated publication. 84 studies were excluded due to lack of clinical effect rate which is the primary outcome evaluated in present study. 4 studies were excluded because the reported groups of participants were same as previous trials. In 108 RCTs, 11 studies were excluded due to other herbal intervention which was combined with SFI as treatment arm. Thus, 97 RCTs [9–20, 22–108] were included for systematic review.

### 3.2. Description of Included Trials

Ninety seven RCTs involved a total of 8,202 patients with HF, including 92 trails (7854 patients) of chronic HF and 5 trials (348 patients) of acute HF. The sample size varied from 24 to 248 participants, with an average of 42 patients per group. Since RCTs of HF on children were excluded, patients are adults (ranged from 28 to 89 years old). More males were included than females (52% males and 48% females). Disease duration was reported in 31 trials, ranging from 3 months to 26 years. 49 trials were observed in inpatients, 5 outpatients [22–26], 5 both inpatients and outpatients [27–31], and 39 unclear. All studies were published in Chinese.

Mortality was reported in eleven studies, while the rest of the eighty eight trials did not mention death. Effect rate was assessed in all the trials, based on the improvement of heart function. Ninety one trials used New York Heart Association (NYHA) Classification of Clinical Status, and six trials used Killip's Rating Standards [22, 25, 26, 33–35] for diagnosing HF and rating the patients. Patients in fifty one trails ranged from II to IV, seven trials II to III, twenty one trials III to IV, and five trials IV according to NYHA Classification; patients in five trials ranged from II to IV and one trial IV according to Killip's Standard*）*.

Results of ultrasonic cardiography were reported in 61 trails (5135 patients) with left ventricular ejection fraction (LVEF) as main parameter. Other parameters such as left ventricular diastolic diameter (LVDd), cardiac output (CO), cardiac index (CI), stroke volume (SV), and A peak E-wave velocity ratio (E/A) were reported in 16, 17, 20, 18, and 11 trials, respectively. N-terminal pro-B-type nature tripeptide (NT-proBNP) level in blood was reported in 12 studies of 887 patients, and 6-minute walk distance (6-MWD) was reported in 8 trials of 630 patients. Heart rate, systolic blood pressure (SBP), and diastolic blood pressure (DBP) were reported in 27, 15, and 13 trials, respectively (Table 1).

### 3.3. Methodological Quality of Included Trials

**Figure 3:**
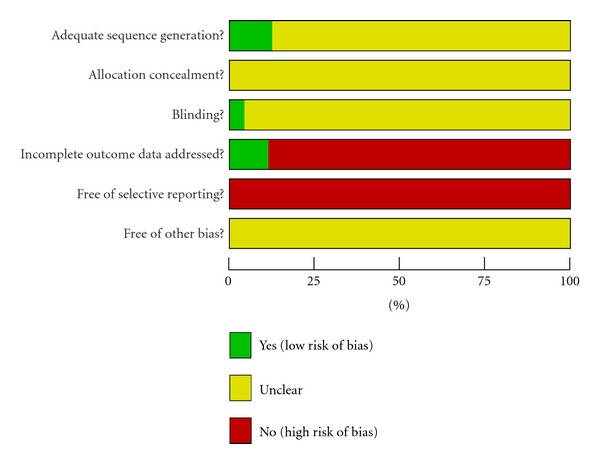
Risk of bias graph: review authors' judgements about each risk of bias item presented as percentages across all included studies.

According to our predefined quality assessment criteria, all of 97 included trials were evaluated as having unclear risk of bias (Table 2, Figure 3). None of the 97 trials reported sample size calculation. Eleven trials described randomization procedures, nine trials [9–11, 20, 30, 38–41] used a random number table, one drew lots [19], and one trial separated patients by odd and even number of patient ID as a quasirandomization [42]. Only one trial [43] blinded both patients and outcome assessors, and three trials [44–46] blinded patients. None of the trials reported adequate allocation concealment. Five out of ninety seven trials mentioned that followup ranged from 3 months to 12 months after treatment. One trial [47] followed all the patients for 12 months, one trail [38] for 6 month, and the rest [9, 11, 12] for 3 months. However, neither of them used intention to treat method.

### 3.4. Effect of the Interventions

The primary outcomes were effect rate and mortality. Secondary outcome measures included LVEF, LVDd, SV, CO, CI, HR, systolic blood pressure (SBP), diastolic blood pressure (DBP), NT-proBNP, and 6-MWD.

#### 3.4.1. Primary Outcomes

**Effect Rate**

**Figure 4:**
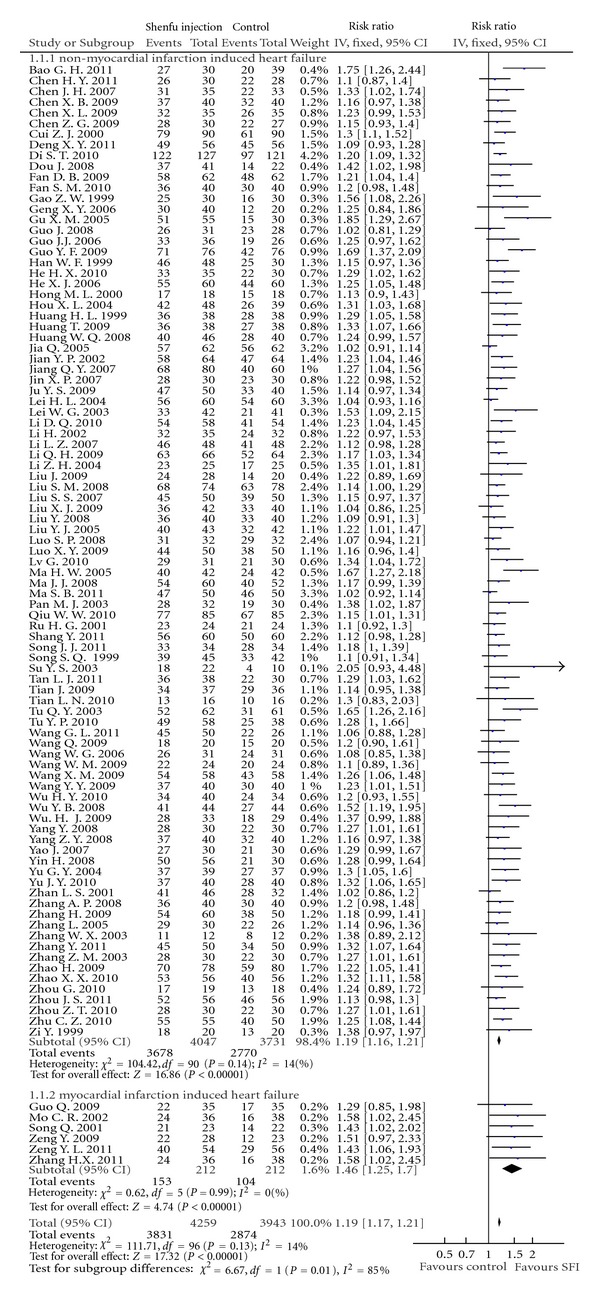
Forest plot of comparison: effect rate.

All the trials reported clinical effect rate to evaluate the outcome, which was based on NYHA Classification of Clinical Status and Killip's Rating Standards. Killip's Rating Standards were used by six trials with patients of myocardial infarction-induced HF, while other trials used NYHA Classification. Most of trails used three categories to evaluate treatment effect including markedly effective (an improvement of two classes on the classification), effective (an improvement of one class), and ineffective (no improvement, deterioration or death), and others only reported total effect. Total effect rate is the combination of markedly effect rate and effect rate. Trials of myocardial infarction-induced HF and nonmyocardial infarction-induced HF were separated into two subgroups. The meta-analysis showed a total significant difference between SFI and control groups on total effect rate (RR: 1.19, 95% CI [1.17, 1.21]; *P* < 0.01). And significant difference appeared in both subgroups separately, with RR ratio 1.19 in subgroup of myocardial infarction-induced HF (95% CI [1.16, 1.21]; *P* < 0.01), and 1.46 in the other subgroup (95% CI [1.25, 1.70]; *P* < 0.01) (Figure 4).

**Death**

**Figure 5:**
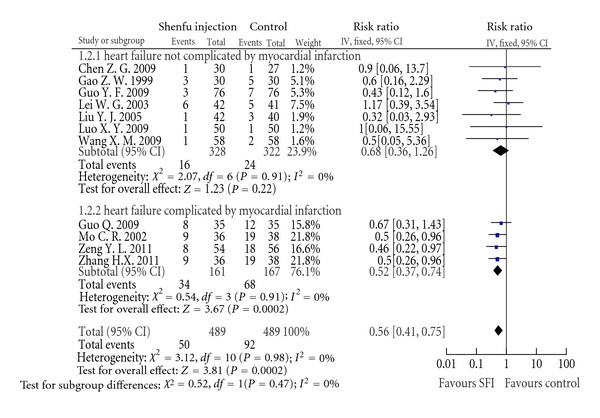
Forest plot of comparison: death.

Eleven studies reported mortality data, and total death number was 142 out of 978. Two trials [12, 38] assessed the mortality with 3- and 6-month followup, respectively, and other trials reported death at the end of treatment course. Trials were also separated into two subgroups depending on whether HF was induced by myocardial infarction. The result of meta-analysis indicated that SFI can significantly reduce mortality of patients of myocardial infarction-induced HF (RR: 0.52, 95% CI [0.37, 0.74]; *P* < 0.01). In the other subgroup, there was no significant difference between mortalities of SFI group and control group (RR: 0.68, 95% CI [0.36, 1.26]; *P* = 0.22). However, total result of both subgroups showed significant difference (RR: 0.56, 95% CI [0.41, 0.75]; *P* < 0.01) (Figure 5).

#### 3.4.2. Secondary Outcomes

**NT-proBNP**

**Figure 6:**
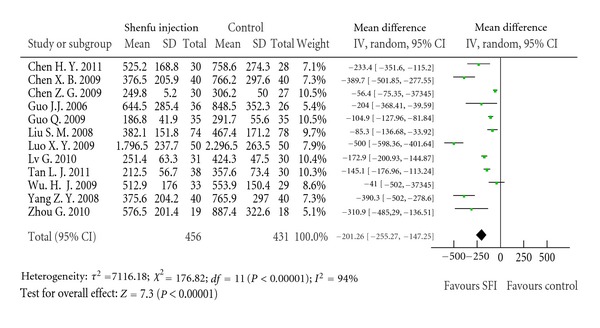
Forest plot of comparison: NT-proBNP.

NT-proBNP level is used for screening and diagnosis of acute HF and may be useful to establish prognosis in HF, as it is typically higher in patients with worse outcome [109]. It was reported in 12 studies [20, 22, 38, 45, 49, 52, 54–59] on 887 patients. Consistent with effect rate and other outcomes, NT-proBNP levels of SFI group were significantly lower than control group (WMD: −201.26; 95% CI [−255.27, − 147.25], *P* < 0.01) (Figure 6).

**6-MWD**

**Figure 7:**
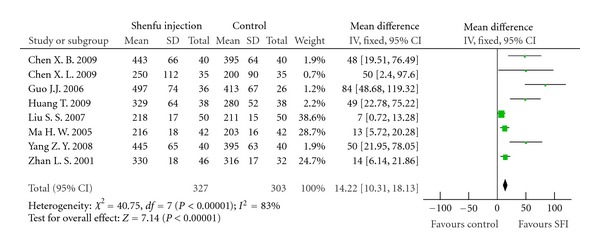
Forest plot of comparison: 6-MWD.

Eight trials [47–54] assessed 6-MWD of patients who received SFI or routine treatment. At the end of treatment, eight trails all showed significant increase in walking distance in SFI group, and meta-analysis result was WMD: 14.22; 95% CI [10.31, 18.13], *P* < 0.01 (Figure 7).

**Heart Rate and Blood Pressure**

Heart rate and blood pressure were reported in 27 and 15 trials, respectively. Meta-analysis showed that there was statistical significance between SFI group and control group (WMD: 6.31; 95% CI [5.18, 7.44], *P* < 0.01) (see Supplementary Figure  1 in Supplementary Material available online at doi: 10.1155/2012/713149). However, there was no significant difference between both SBP and DBP in two groups (WMD: −0.07; 95% CI [−0.42, 0.27], *P* = 0.68) (WMD: −0.37; 95% CI [−0.97, 0.23], *P* = 0.22) (Supplementary Figures 2 and 3 in Supplementary Material available online at doi: 10.1155/2012/713149).

**Results of Ultrasonic Cardiography**

LVEF is the ratio of the stroke volume and the left ventricular end-diastolic volume [107]. It is usually used for the assessment of HF and drug efficacy. Sixty-one studies reported the outcomes for LVEF. Meta-analysis showed that SFI group was better than control group in increasing LVEF (WMD: 6.31; 95% CI [5.18, 7.44], *P* < 0.01) (Supplementary Figure  4).

SV is the volume per stroke by left ventricle, and CO is the volume of blood being pumped by the heart in the time interval of one minute [107]. CI is a vasodynamic parameter that is relating CO to body surface area [107]. All the three parameters indicate left ventricular systolic function, as LVEF does. This paper made meta-analysis of these outcomes, respectively; results showed that SFI group was better than control group in these three parameters: SV (WMD: 7.25; 95% CI [4.60, 9.90], *P* < 0.01); CO (WMD: 0.67; 95% CI [0.47, 0.87], *P* < 0.01); CI (WMD: 0.36; 95% CI [0.23, 0.48], *P* < 0.01) (Supplementary Figures  5–7).

E/A ratio is widely accepted as a clinical marker of diastolic HF, and E/A ratio is reduced in diastolic dysfunction [108]. The result of meta-analysis of E/A ratio was WMD: 0.15; 95% CI [0.08, 0.22], *P* < 0.01, which indicated that SFI better improved diastolic function of heart on HF patients than conventional medicine treatment did (Supplementary Figure  8).

LVDd is the end-diastolic dimension of the left ventricle. There was no statistical significance between SFI combined with conventional medicine treatment and conventional medicine treatment groups (WMD: −1.59; 95% CI [−5.29, 2.12], *P* = 0.40) (Supplementary Figure  9).

#### 3.4.3. Quality of Life

 None of the trials reported quality of life.

### 3.5. Publication Bias

**Figure 8:**
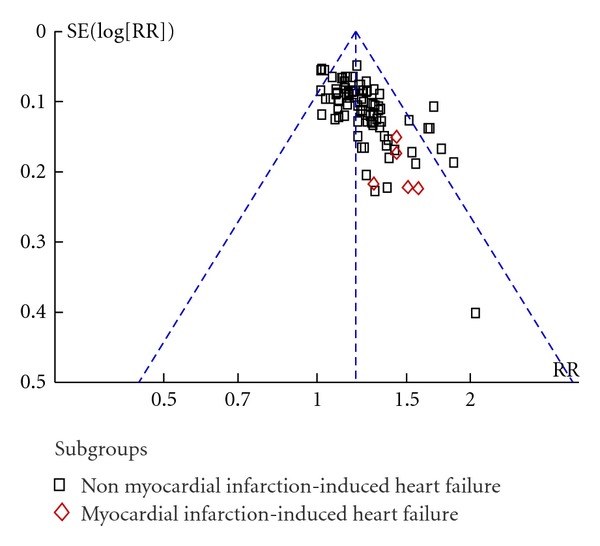
Funnel plot of comparison: effect rate.

Funnel plots based on the data of effect rate were elaborated in Figure 8. The figure was asymmetrical, which indicated that potential publication bias might influence the results of this paper. Although we conducted comprehensive searches and tried to avoid bias, since all trials were published in Chinese, we could not exclude potential publication bias.

### 3.6. Adverse Effect

Thirty seven out of ninety seven trials mentioned the adverse effect except in sixty-two trials which was unclear. Thirteen trials [9–20, 60] reported the following thirteen specific symptoms of side effects including dry mouth, dryness heat, fullness of the head, insomnia, dysphoria, skin itching, tachycardia, feverish dysphoria, flushing of face, tidal fever, dizziness due to low blood pressure, gastrointestinal discomfort, and palpitation. Among these side effects, dry mouth and fullness of the head were reported in 4 trails with 14 and 10 cases, respectively. These symptoms were regarded to be mild and recovered spontaneously after SFI withdrawal. Twenty four trials reported that no side effects were observed in the SFI group (Table 3).

The above side effects might be related to higenamine, which is the active ingredient of prepared aconite root. In TCM books and papers, prepared aconite root is frequently mentioned with adverse effects as dry mouth, dryness heat, fullness of the head, and dysphoria due to its strong effect of strengthening* yang*.

## 4. Discussion

In many years, western medicine has made tremendous progress and has become the dominating medical treatment worldwide. However, it has been increasingly recognized that western medicine may sometimes fail to treat an illness, whereas such illness is reportedly improved by the so-called complementary medicine based on a different theory [110, 111]. Although conventional therapeutic approaches were used in HF, it remained a cardiovascular disease with an increasing hospitalization burden and an ongoing drain on health care expenditures [2]. TCM plays an important role in treating HF in China. SFI was a traditional Chinese Patent Medicine based on TCM theory, which was approved by the Chinese State Food and Drug Administration. In recent 10 years, it has been widely used for HF in many hospitals and clinics. However, few RCTs of SFI were reported in English journals, and it was difficult for western doctors to accept SFI as an alternative medicine. Although there were two systematic reviews about SFI for HR published in Chinese journal [112, 113], only 16 and 8 trials were included in their study. Therefore, the present study aimed to systematically assess the efficacy and safety of SFI for HR. 

Data from the 97 RCTs demonstrated that SFI combined with conventional medication may be more effective on HF than conventional medication only. With improvement of cardiofunction of patients, based on NYHA Classification of Clinical Status and Killip's Rating Standards, the effect rate of SFI group was, on average, 17 percent more than control group (RR, 1.19; 95% CI, 1.17 to 1.21). Mortality data was another primary outcome. In eleven trials in which death was recorded, meta-analysis showed that mortality was significantly lower in SFI group than control group. This result was mainly contributed by subgroup of HF induced by myocardial infarction, for patients in this subgroup were more vulnerable.

Ultrasonic cardiography is widely used in inspection for HF patients. From results of ultrasonic cardiography, the systolic and diastolic functions of heart can be interpreted. LVEF, CO, CI, SV, LVDd, and E/A were reviewed by us, respectively. There was significant difference between SFI group and control group in all of the outcomes except LVDd. Since SV, CO, CI, and LVEF indicate heart systolic function, and E/A indicate heart diastolic function, conclusion can be drawn that SFI benefits both systolic and diastolic functions of heart. But it did not have significant effect on expansion of heart. NT-proBNP level in serum of SFI group was significantly lower than the control group, which is inconsistent with effect rate. 6-MWD results of patients of SFI group also are better than thos of control group. It indicates that SFI had a tendency to improve life status. Furthermore, heart rate was obviously reduced in SFI group, which could be related to alleviation of HF.

Meta-analysis on LVEF, CO, CI, SV, LVDd, E/A, heart rate, and NT-proBNP all showed significant heterogeneity. Several possible explanations can be given, for example, different complications, different instruments employed for test, and difference in methodological rigor.

 However, we should consider the following limitations before accepting the findings of this paper.

Firstly, the methodological quality of the included studies is generally poor. Although all trials claimed to perform randomization, only eleven trials reported the procedure to generate the sequence, while the rest of trials did not give any details of the randomization method. Thus, whether randomization was effectively conducted in these trials was doubtful. Blinding was mentioned in four trials, with one trial blinded patients and outcome assessors [43] and three blinded patients only [44–46]. Neither of them described the methods of allocation concealment. Dropouts account and intention to treat analysis were not mentioned in all the trails. Due to inadequate reporting of methodological design, it was possible that there was performance bias and detection bias due to patients and researchers being aware of the therapeutic interventions for the subjective outcome measures. Therefore, we cannot draw a confident conclusion that there were significant beneficial effects of SFI combined with conventional medicine treatment compared with conventional medicine treatment.

Secondly, limited outcomes were reported, especially death and adverse events. Since HF is a disease with high mortality, death is the most important primary outcome. However, only eleven studies out of ninety seven trials reported death, and most of the eleven trials assessed mortality at the end of treatment, without followup. Another outcome was adverse events, to which more attention should be attached. Only 37.4% of the trials described the occurrence of adverse events, indicating an incomplete evaluation of the safety profile of SFI, as well as poor quality of reporting. In most trials, the duration of therapy and followup was too short to achieve conclusive results, except that only one trial had a treatment of 10 months [47]. Only 6 included trials had a followup period (ranged from 3 to 12 months), while in rest of studies, the outcomes were evaluated at the end of the treatment (mostly range from 14 to 21 days). In order to evaluate drug efficacy for chronic HF, long-term improvement (at least 6 months) of chronic HF-specific clinical symptoms is needed [114], because some drugs have shown to increase mortality in the long-term application despite a short-term improvement in clinical symptoms [115]. In addition, long-term toxicity assessment was also important for drug safety evaluation.

Next, although irrespective of languages, all the trials included in this paper were published in Chinese journals, Zhang et al. and Liu et al. [115, 116] found that some Asian countries including China unusually publish high proportions of positive results. Wu et al. [117] and Jin et al. [118] accounted that RCTs in Chinese journals often had problems of low methodological quality and selective publication of positive results. Considering that all of the ninety seven trials were published in Chinese, the publication bias possibly existed. 

Additionally, none of the ninety seven trials reported sample size calculation, and in most trials, the sample size was limited. Further high-quality studies with larger sample size are needed to confirm the effectiveness of SFI in treating HF. Quality of life was not reported in all the including trials. Although 6-MWD showed a tendency of SFI to improve life status for HF patients, we advise future RCTs to select outcomes of life quality according to international practice.

Considering that there was no sufficient amount of high-quality trials on SFI treating patients with HF, the effectiveness and safety of SFI need further rigorous trials to prove, which should be consistent with the CONSORT statement on the reporting of the results of randomized trials (http://www.consort-statement.org/).

## 5. Conclusion

The preliminary conclusion of the current study suggests that SFI might be beneficial to patients with HF. More rigorously designed trails with high methodological quality are necessary for further proof.

## Supplementary Material

Compared with routine treatment and/or device support, SFI combined with routine treatment and/or device support showed better effect on ultrasonic cardiography. Significance are showed in most parameters of cardiography, namely, LVEF (WMD: 6.31; 95% CI [5.18, 7.44], P<0.01), SV (WMD:7.25; 95%CI [4.60, 9.90], P<0.01), CO (WMD: 0.67; 95%CI [0.47, 0.87], P<0.01), CI (WMD: 0.36; 95%CI [0.23, 0.48], P<0.01) and E/A ratio (WMD:0.15; 95%CI [0.08, 0.22], P<0.01). There were no significant difference in LVDd (WMD: −1.59; 95% CI [-5.29, 2.12], P=0.40), systolic blood pressure (WMD: −0.07; 95%CI [−0.42, 0.27], P=0.68) and diastolic blood pressure (WMD: −0.37; 95% CI [−0.97, 0.23], P=0.22) between SFI and routine treatment groups.

## Figures and Tables

**Table 1:** Characters of including trials.

Author Name	Inpatient (Y/N)	Course	Experiment group	Control group	NYHA classification	Disease duration	Followup (month)
Bao and Yu [61]	Y	14 d	Conventional medicine treatment plus SFI 50 mL, qd, iv.gtt	Conventional medicine treatment	II–IV	Unclear	No
Chen [55]	Unclear	14 d	Conventional medicine treatment plus SFI 60 mL, qd, iv.gtt	Conventional medicine treatment	III-IV	Unclear	No
Chen and Liu [14]	Y	60 d	Conventional medicine treatment plus SFI 50 mL, qd, iv.gtt plus metoprolol 6.25 mg, bid, po	Conventional medicine treatment plus metoprolol 6.25 mg, bid,po	II-III	1–15 y	No
Chen and Li [51]	Y	14 d	Conventional medicine treatment plus SFI 60 mL, qd, iv.gtt plus sodium nitroprusside 50 mg, iv.gtt	Conventional medicine treatment plus sodium nitroprusside 50 mg, iv.gtt	IV	Unclear	No
Chen et al. [52]	Y	15 d	Conventional medicine treatment plus SFI 60 mL, qd, iv.gtt	Conventional medicine treatment	II–IV	4.5 y on average	No
Chen et al. [56]	Y	14 d	Conventional medicine treatment plus SFI 50 mL, qd, iv.gtt	Conventional medicine treatment	III-IV	1.5 month–8 y	No
Cui [86]	Unclear	10 d	Conventional medicine treatment plus SFI 50 mL, qd, iv.gtt	Digoxigenin 0.25 mg	II-III	2–7 y	No
Deng and Tang [15]	Y	14 d	Conventional medicine treatment plus SFI 20–40 mL, qd, iv.gtt	Conventional medicine treatment	II–IV	Unclear	No
Di [67]	Unclear	Unclear	Conventional medicine treatment plus SFI 40 mL, bid, iv.gtt	Conventional medicine treatment	II–IV	3–17 y	No
Dou [97]	Unclear	10 d	Conventional medicine treatment plus SFI 50 mL, qd, iv.gtt	Conventional medicine treatment	II–IV	12 ± 1.5 y	No
Fan [60]	Y	21 d	Conventional medicine treatment plus SFI 60 mL, qd, iv.gtt	Metoprolol 12.5 mg, bid, po, +captopril 12.5 mg, tid, po	II–IV	Unclear	No
Fan et al. [101]	Unclear	14 d	Conventional medicine treatment plus SFI 40 mL, qd, iv.gtt	Conventional medicine treatment	II–IV	Unclear	No
Geng et al. [27]	Both	12 d	Conventional medicine treatment plus SFI 60 mL, qd, iv.gtt	Conventional medicine treatment	III-IV	0.5–9 y	No
Gu et al. [69]	Y	14 d	Conventional medicine treatment plus SFI 100 mL, qd, iv.gtt	Conventional medicine treatment	II–IV	1.5–12 y	No
Guo et al. [49]	Unclear	14 d	Conventional medicine treatment plus SFI 60 mL, qd, iv.gtt	Conventional medicine treatment	III-IV	Unclear	No
Guo et al. [23]	N	7 d	Conventional medicine treatment plus SFI 20 mL, iv + 50 mL, qd, iv.gtt plus non invasive positive pressure ventilation	Conventional medicine treatment plus non invasive positive pressure ventilation	Unclear	Unclear	No
Guo et al. [102]	Y	7 d	Conventional medicine treatment plus SFI 40–60 mL, qd, iv.gtt plus invasive respiratory support	Conventional medicine treatment plus invasive respiratory support	IV	Unclear	No
Han and Li [36]	Y	15 d	Conventional medicine treatment plus SFI 50 mL, qd, iv.gtt	Conventional medicine treatment	III-IV	4.54 ± 2.1 y	No
He [70]	Unclear	14 d	Conventional medicine treatment plus SFI 40 mL, qd, iv.gtt	Conventional medicine treatment	II–IV	1–14 y	No
He [98]	Unclear	7–20 d/10–30 d	Conventional medicine treatment plus SFI 20–40 mL, qd, iv.gtt	Conventional medicine treatment	III-IV	3–16 y	No
Hong [44]	Unclear	14 d	Conventional medicine treatment plus SFI 80 mL, qd, iv.gtt	Conventional medicine treatment	III-IV	Unclear	No
Hou and Hong [17]	Unclear	7 d	Conventional medicine treatment plus SFI 60–100 mL, qd, iv.gtt	Conventional medicine treatment	II–IV	Unclear	No
Huang [13]	Unclear	7 d	Conventional medicine treatment plus SFI 20 mL iv + 40 mL, qd, iv.gtt	Conventional medicine treatment	III-IV	Unclear	No
Huang [53]	Y	14 d	Conventional medicine treatment plus SFI 40 mL, qd, iv.gtt	Conventional medicine treatment	II–IV	Unclear	No
Huang et al. [24]	N	Unclear	Conventional medicine treatment plus SFI 50 mL, qd, iv.gtt plus sodium nitroprusside 50 mg, iv.gtt	Conventional medicine treatment plus sodium nitroprusside 50 mg, iv.gtt	Unclear	Unclear	No
Jia and Yang [71]	Y	20 d	Conventional medicine treatment plus SFI 40 mL, qd, iv.gtt	Conventional medicine treatment	II–IV	Unclear	No
Jian and Chen [88]	Unclear	14 d	Conventional medicine treatment plus SFI 60–80 mL, bid, iv.gtt	Conventional medicine treatment	III-IV	6.5 y on average	No
Jiang [62]	Y	14 d	Conventional medicine treatment plus SFI 50 mL, qd, iv.gtt	Conventional medicine treatment	II–IV	Unclear	No
Jin and Guo [95]	Y	14 d	Conventional medicine treatment plus SFI 50 mL, qd, iv.gtt	Conventional medicine treatment	II–IV	Unclear	No
Ju [37]	Unclear	14 d	Conventional medicine treatment plus SFI 30 mL, qd, iv.gtt	Conventional medicine treatment	II–IV	Unclear	No
Lei and Li [92]	Y	7–10 d	Conventional medicine treatment plus SFI 50 mL, qd, iv.gtt	Conventional medicine treatment	II–IV	Unclear	No
Lei et al. [12]	Y	14 d	Conventional medicine treatment plus SFI 50 mL, qd, iv.gtt	Conventional medicine treatment	II–IV	1–18 y	3
Li et al. [9]	Y	14 d	Conventional medicine treatment plus SFI 50 mL, qd, iv.gtt	Conventional medicine treatment	II–IV	1–20 y	3
Li et al. [72]	Y	15 d	Conventional medicine treatment plus SFI 30 mL, qd, iv.gtt	Conventional medicine treatment	III-IV	5–26 y	No
Li [96]	Unclear	15 d	Conventional medicine treatment plus SFI 40 mL, qd, iv.gtt	Conventional medicine treatment	III-IV	Unclear	No
Li et al. [73]	Unclear	15 d	Conventional medicine treatment plus SFI 100 mL, qd, iv.gtt plus sodium nitroprusside 50 mg	Conventional medicine treatment plus sodium nitroprusside 50 mg	IV	l–25 y	No
Li [93]	Unclear	10 d	Conventional medicine treatment plus SFI 1 mL/kg body weight, qd, iv.gtt	Conventional medicine treatment	II–IV	Unclear	No
Liu [75]	Y	21 d	Conventional medicine treatment plus SFI 40 mL, qd, iv.gtt	Conventional medicine treatment	II–IV	Unclear	No
Liu and Sun [18]	Unclear	7 d	Conventional medicine treatment plus SFI 100 mL, qd, iv.gtt	Conventional medicine treatment	Unclear	Unclear	No
Liu and Chan [50]	Unclear	14 d	Conventional medicine treatment plus SFI 50 mL, qd, iv.gtt	Conventional medicine treatment	II–IV	Unclear	No
Liu et al. [20]	Y	28 d	Conventional medicine treatment plus SFI 40 mL, qd, iv.gtt	Conventional medicine treatment	II–IV	9 month–14 y	No
Liu [74]	Y	14 d	Conventional medicine treatment plus SFI 50 mL, qd, iv.gtt	Conventional medicine treatment	II–IV	Unclear	No
Liu et al. [94]	Unclear	14 d	Conventional medicine treatment plus SFI 40 mL, qd, iv.gtt	Conventional medicine treatment	II–IV	Unclear	No
Lv [57]	Y	14 d	Conventional medicine treatment plus SFI 50 mL, qd, iv.gtt	Conventional medicine treatment	III-IV	Unclear	No
Luo et al. [76]	Y	14 d	Conventional medicine treatment plus SFI 50 mL, qd, iv.gtt	Conventional medicine treatment plus sodium nitroprusside	III-IV	Unclear	No
Luo et al. [38]	Unclear	10 d/m 6 months	Conventional medicine treatment plus SFI 60 mL, qd, iv.gtt	Conventional medicine treatment	II–IV	>3 months	6
Ma et al. [48]	Unclear	20 d	Conventional medicine treatment plus SFI 30–40 mL, qd, iv.gtt	Conventional medicine treatment	II-III	Unclear	No
Ma and Huang [99]	Y	14 d	Conventional medicine treatment plus SFI 60 mL, qd, iv.gtt	Conventional medicine treatment	II–IV	Unclear	No
Ma [77]	Unclear	15 d	Conventional medicine treatment plus SFI 20 mL, qd, iv.gtt	Conventional medicine treatment	II–IV	Unclear	No
Pan et al. [89]	Y	14 d	Conventional medicine treatment plus SFI 100 mL, qd, iv.gtt	Conventional medicine treatment plus dobutamine hydrochloride 40 ng, qd, iv.gtt	II–IV	2.5 month–11 y	No
Qiu [103]	Y	14 d	Conventional medicine treatment plus SFI 50 mL, qd, iv.gtt	Conventional medicine treatment	II–IV	Unclear	No
Ru [46]	Unclear	10 d	Conventional medicine treatment plus SFI 60 mL, qd, iv.gtt	Conventional medicine treatment	III-IV	Unclear	No
Shang [78]	Y	14 d	Conventional medicine treatment plus SFI 50 mL, qd, iv.gtt	Conventional medicine treatment	II–IV	Unclear	No
Song [106]	Y	15 d	Conventional medicine treatment plus SFI 50 mL, qd, iv.gtt	Conventional medicine treatment	II-III	Unclear	No
Song et al. [10]	Y	15 d	Conventional medicine treatment plus SFI 60 mL, qd, iv.gtt	Conventional medicine treatment	II–IV	Unclear	No
Su [90]	Y	14 d	Conventional medicine treatment plus SFI 100 mL, qd, iv.gtt	Conventional medicine treatment	II–IV	Unclear	No
Tan et al. [58]	Unclear	14 d	Conventional medicine treatment plus SFI 60 mL, qd, iv.gtt	Conventional medicine treatment	II–IV	Unclear	No
Tian and Gong [16]	Y	14 d	Conventional medicine treatment plus SFI 60 mL, qd, iv.gtt	Conventional medicine treatment	II–IV	2–20 y	No
Tian [80]	Y	15 d	Conventional medicine treatment plus SFI 50 mL, qd, iv.gtt	Conventional medicine treatment	III-IV	>7 months	No
Tu and Yang [63]	Y	14 d	Conventional medicine treatment plus SFI 80 mL, qd, iv.gtt	Conventional medicine treatment	II–IV	Unclear	No
Tu et al. [32]	Unclear	14 d	Conventional medicine treatment plus SFI 100 mL, qd, iv.gtt	Conventional medicine treatment	II–IV	Unclear	No
G. L. Wang and J. Wang [104]	Y	15 d	Conventional medicine treatment plus SFI 60 mL, qd, iv.gtt	Conventional medicine treatment	III-IV	2.5–16 y	No
Wang [100]	Y	14 d	Conventional medicine treatment plus SFI 40 mL, qd, iv.gtt	Conventional medicine treatment	Unclear	22.3 ± 4.8 y	No
Wang [39]	Unclear	15 d	Conventional medicine treatment plus SFI 40 mL, qd, iv.gtt	Conventional medicine treatment	II–IV	Unclear	No
Wang [28]	Both	14 d	Conventional medicine treatment plus SFI 60 mL, qd, iv.gtt	Conventional medicine treatment	II–IV	0.6–7 y	No
Wang and Ye [87]	Y	10 d	Conventional medicine treatment plus SFI 40–100 mL, qd, iv.gtt	Conventional medicine treatment	III-IV	14.2 y mean	No
Wang et al. [81]	Y	14 d	Conventional medicine treatment plus SFI 50 mL, qd, iv.gtt	Conventional medicine treatment	IV	3–10 y	No
Wu and Duan [45]	Y	14 d	Conventional medicine treatment plus SFI 50 mL, qd, iv.gtt	Conventional medicine treatment	II-III	Unclear	No
Wu and Wang [64]	Y	14 d	Conventional medicine treatment plus SFI 100 mL, qd, iv.gtt	Conventional medicine treatment	II–IV	Unclear	No
Wu et al. [40]	Unclear	10 d	Conventional medicine treatment plus SFI 50 mL, qd, iv.gtt	Conventional medicine treatment	II–IV	Unclear	No
Yang and Wu [82]	Y	14 d	Conventional medicine treatment plus SFI 60 mL, qd, iv.gtt	Conventional medicine treatment	III-IV	Unclear	No
Yang et al. [54]	Y	15 d	Conventional medicine treatment plus SFI 50 mL, qd, iv.gtt	Conventional medicine treatment	II–IV	5.1 y	No
Yao and Lu [65]	Y	14 d	Conventional medicine treatment plus SFI 50 mL, qd, iv.gtt	Conventional medicine treatment	II–IV	Unclear	No
Yin [83]	Y	14 d	Conventional medicine treatment plus SFI 40 mL, qd, iv	Conventional medicine treatment	II–IV	0.5–12 y	No
Yu et al. [84]	Unclear	14 d	Conventional medicine treatment plus SFI 50 mL, qd, iv.gtt	Conventional medicine treatment	II–IV	Unclear	No
Yu [66]	Unclear	14 d	Conventional medicine treatment plus SFI 50 mL, qd, iv.gtt	Conventional medicine treatment	II–IV	Unclear	No
Zhan and Yang [47]	Unclear	20 d	Conventional medicine treatment plus SFI 40 mL, qd, iv.gtt plus metoprolol. 25 mg–75 mg, bid po	Conventional medicine treatment plus metoprolol. 25 mg–75 mg, bid, po	II-III	Unclear	12
Zhang [79]	Y	14 d	Conventional medicine treatment plus SFI 50 mL, qd, iv.gtt	Conventional medicine treatment	II–IV	3–15 y	No
Zhang et al. [85]	Y	20 d	Conventional medicine treatment plus SFI 30 mL, qd, iv.gtt	Conventional medicine treatment	II–IV	Unclear	No
Zhang [42]	Y	21 d	Conventional medicine treatment plus SFI 60 mL, qd, iv.gtt	Conventional medicine treatment	II-III	Unclear	No
Zhang and Pan [30]	Both	14 d	Conventional medicine treatment plus SFI 40–60 mL, qd, iv.gtt	Conventional medicine treatment	III-IV	2–16 y	No
Zhang [29]	Both	14 d	Conventional medicine treatment plus SFI 60 mL, qd, iv.gtt	Conventional medicine treatment	III-IV	Unclear	No
Zhang [43]	Y	14 d	Conventional medicine treatment plus SFI 50 mL, qd, iv.gtt	Conventional medicine treatment	II–IV	Unclear	No
Zhao et al. [11]	Y	14 d	Conventional medicine treatment plus SFI 50 mL, qd, iv.gtt	Conventional medicine treatment plus isoket 10 mg,qd,iv.gtt	II–IV	1–20 y	3
Zhao [91]	Y	14 d	Conventional medicine treatment plus SFI 60 mL, qd, iv.gtt	Conventional medicine treatment	Unclear	Unclear	No
Zhou [59]	Y	14 d	Conventional medicine treatment plus SFI 60 mL, qd, iv.gtt	Conventional medicine treatment	IV	3–15 y	No
Zhou [19]	Y	10 d	Conventional medicine treatment plus SFI 80 mL, qd, iv.gtt	Conventional medicine treatment	II–IV	Unclear	No
Zhou et al. [31]	Both	14 d	Conventional medicine treatment plus SFI 50 mL, qd, iv.gtt	Conventional medicine treatment	II–IV	Unclear	No
Zhu and Ma [105]	Y	15 d	Conventional medicine treatment plus SFI 50 mL, qd, iv.gtt	Conventional medicine treatment	III-IV	Unclear	No
Zi and Li [41]	Y	14 d	Conventional medicine treatment plus SFI 40–100 mL, qd, iv or iv.gtt	Conventional medicine treatment plus dobutamine hydrochloride 50–100 mg	II–IV	Unclear	No
Guo et al. [22]	N	14 d	Conventional medicine treatment plus SFI 60–100 mL, bid, iv.gtt	Conventional medicine treatment	II–IV	Unclear	No
Mo and Zhao [25]	N	7 d	Conventional medicine treatment plus SFI 60–100 mL, qd, iv.gtt	Conventional medicine treatment	II–IV	1–7 d	No
Song and Zhang [33]	Y	10 d	Conventional medicine treatment plus SFI 40–60 mL, qd, iv.gtt	Conventional medicine treatment plus dobutamine hydrochloride 40 mg	II–IV	Unclear	No
Zeng et al. [34]	Y	7 d	Conventional medicine treatment plus SFI 50 mL, qd, iv.gtt	Conventional medicine treatment	IV	Unclear	No
Zeng [26]	N	10 d	Conventional medicine treatment plus SFI 60–100 mL, qd, iv.gtt	Conventional medicine treatment	II–IV	1–72 h	No
Zhang [35]	Y	14 d	Conventional medicine treatment plus SFI 60 mL, qd, iv.gtt	Conventional medicine treatment	II–IV	Unclear	No

Conventional medicine treatment includes sitting up position, supplemental oxygen, vasodilator such as nitroglycerine, diuretics such as furosemide, and cardiotonic agents such as lanatoside C, ACE inhibitors, and *β*-blockers.

**Table 2:** Bias of including trails.

Author Name	Sequence generation	Allocation concealment	Blinding	Incomplete outcome data	Selective outcome reporting	Other source of bias	Risk of bias
Bao and Yu [61]	Unclear	Unclear	N	N	N	Unclear	Unclear
Chen [55]	Unclear	Unclear	N	N	N	Unclear	Unclear
Chen and Liu [14]	Unclear	Unclear	N	N	N	Unclear	Unclear
Chen and Li [51]	Unclear	Unclear	N	N	N	Unclear	Unclear
Chen et al. [52]	Unclear	Unclear	N	N	N	Unclear	Unclear
Chen et al. [56]	Unclear	Unclear	N	Y	N	Unclear	Unclear
Cui [86]	Unclear	Unclear	N	N	N	Unclear	Unclear
Deng and Tang [15]	Unclear	Unclear	N	N	N	Unclear	Unclear
Di [67]	Unclear	Unclear	N	N	N	Unclear	Unclear
Dou [97]	Unclear	Unclear	N	N	N	Unclear	Unclear
Fan [60]	Unclear	Unclear	N	N	N	Unclear	Unclear
Fan et al. [101]	Unclear	Unclear	N	N	N	Unclear	Unclear
Gao et al. [68]	Unclear	Unclear	N	N	N	Unclear	Unclear
Geng et al. [27]	Unclear	Unclear	N	N	N	Unclear	Unclear
Gu et al. [69]	Unclear	Unclear	N	N	N	Unclear	Unclear
Guo et al. [49]	Unclear	Unclear	N	N	N	Unclear	Unclear
Guo et al. [23]	Unclear	Unclear	N	N	N	Unclear	Unclear
Guo et al. [102]	Unclear	Unclear	N	Y	N	Unclear	Unclear
Han and Li [36]	Unclear	Unclear	N	N	N	Unclear	Unclear
He [70]	Unclear	Unclear	N	N	N	Unclear	Unclear
He [98]	Unclear	Unclear	N	N	N	Unclear	Unclear
Hong [44]	Unclear	Unclear	Single-blind	N	N	Unclear	Unclear
Hou and Hong [17]	Unclear	Unclear	N	N	N	Unclear	Unclear
Huang [13]	Unclear	Unclear	N	N	N	Unclear	Unclear
Huang [53]	Unclear	Unclear	N	N	N	Unclear	Unclear
Huang et al. [24]	Unclear	Unclear	N	N	N	Unclear	Unclear
Jia and Yang [71]	Unclear	Unclear	N	N	N	Unclear	Unclear
Jian and Chen [88]	Unclear	Unclear	N	N	N	Unclear	Unclear
Jiang [62]	Unclear	Unclear	N	N	N	Unclear	Unclear
Jin and Guo [95]	Unclear	Unclear	N	N	N	Unclear	Unclear
Ju [37]	Unclear	Unclear	N	N	N	Unclear	Unclear
Lei and Li [92]	Unclear	Unclear	N	N	N	Unclear	Unclear
Lei et al. [12]	Unclear	Unclear	N	Y	N	Unclear	Unclear
Li et al. [9]	Random number table	Unclear	N	N	N	Unclear	Unclear
Li et al. [72]	Unclear	Unclear	N	N	N	Unclear	Unclear
Li [96]	Unclear	Unclear	N	N	N	Unclear	Unclear
Li et al. [73]	Unclear	Unclear	N	N	N	Unclear	Unclear
Li [93]	Unclear	Unclear	N	N	N	Unclear	Unclear
Liu [75]	Unclear	Unclear	N	N	N	Unclear	Unclear
Liu and Sun [18]	Unclear	Unclear	N	N	N	Unclear	Unclear
Liu and Chan [50]	Unclear	Unclear	N	N	N	Unclear	Unclear
Liu et al. [20]	Random number table	Unclear	N	Y	N	Unclear	Unclear
Liu [74]	Unclear	Unclear	N	Y	N	Unclear	Unclear
Liu et al. [94]	Unclear	Unclear	N	N	N	Unclear	Unclear
Lv [57]	Unclear	Unclear	N	N	N	Unclear	Unclear
Luo et al. [76]	Unclear	Unclear	N	N	N	Unclear	Unclear
Luo et al. [38]	Random number table	Unclear	N	Y	N	Unclear	Unclear
Ma et al. [48]	Unclear	Unclear	N	N	N	Unclear	Unclear
Ma and Huang [99]	Unclear	Unclear	N	N	N	Unclear	Unclear
Ma [77]	Unclear	Unclear	N	N	N	Unclear	Unclear
Pan et al. [89]	Unclear	Unclear	N	N	N	Unclear	Unclear
Qiu [103]	Unclear	Unclear	N	N	N	Unclear	Unclear
Ru [46]	Unclear	Unclear	Single-blind	N	N	Unclear	Unclear
Shang [78]	Unclear	Unclear	N	N	N	Unclear	Unclear
Song [106]	Unclear	Unclear	N	N	N	Unclear	Unclear
Song et al. [10]	Random number table	Unclear	N	N	N	Unclear	Unclear
Su [90]	Unclear	Unclear	N	N	N	Unclear	Unclear
Tan et al. [58]	Unclear	Unclear	N	N	N	Unclear	Unclear
Tian and Gong [16]	Unclear	Unclear	N	N	N	Unclear	Unclear
Tian [80]	Unclear	Unclear	N	N	N	Unclear	Unclear
Tu and Yang [63]	Unclear	Unclear	N	N	N	Unclear	Unclear
Tu et al. [32]	Unclear	Unclear	N	N	N	Unclear	Unclear
G. L. Wang and J. Wang [104]	Unclear	Unclear	N	N	N	Unclear	Unclear
Wang [100]	Unclear	Unclear	N	N	N	Unclear	Unclear
Wang [39]	Random number table	Unclear	N	N	N	Unclear	Unclear
Wang [28]	Unclear	Unclear	N	N	N	Unclear	Unclear
Wang and Ye [87]	Unclear	Unclear	N	Y	N	Unclear	Unclear
Wang et al. [81]	Unclear	Unclear	N	N	N	Unclear	Unclear
Wu and Duan [45]	Unclear	Unclear	Single-blind	Y	N	Unclear	Unclear
Wu and Wang [64]	Unclear	Unclear	N	N	N	Unclear	Unclear
Wu et al. [40]	Random number table	Unclear	N	N	N	Unclear	Unclear
Yang and Wu [82]	Unclear	Unclear	N	N	N	Unclear	Unclear
Yang et al. [54]	Unclear	Unclear	N	N	N	Unclear	Unclear
Yao and Lu [65]	Unclear	Unclear	N	N	N	Unclear	Unclear
Yin [83]	Unclear	Unclear	N	N	N	Unclear	Unclear
Yu et al. [84]	Unclear	Unclear	N	N	N	Unclear	Unclear
Yu [66]	Unclear	Unclear	N	N	N	Unclear	Unclear
Zhan and Yang [47]	Unclear	Unclear	N	Y	N	Unclear	Unclear
Zhang [79]	Unclear	Unclear	N	N	N	Unclear	Unclear
Zhang et al. [85]	Unclear	Unclear	N	N	N	Unclear	Unclear
Zhang [42]	odd and even number of ID	Unclear	N	N	N	Unclear	Unclear
Zhang and Pan [30]	Unclear	Unclear	N	N	N	Unclear	Unclear
Zhang [29]	Random number table	Unclear	N	N	N	Unclear	Unclear
Zhang [43]	Unclear	Unclear	Double-blind	N	N	Unclear	Unclear
Zhao et al. [11]	Random number table	Unclear	N	N	N	Unclear	Unclear
Zhao [91]	Unclear	Unclear	N	N	N	Unclear	Unclear
Zhou [59]	Unclear	Unclear	N	N	N	Unclear	Unclear
Zhou [19]	Drew lots	Unclear	N	N	N	Unclear	Unclear
Zhou et al. [31]	Unclear	Unclear	N	N	N	Unclear	Unclear
Zhu and Ma [105]	Unclear	Unclear	N	N	N	Unclear	Unclear
Zi and Li [41]	Random number table	Unclear	N	N	N	Unclear	Unclear
Guo et al. [22]	Unclear	Unclear	N	Y	N	Unclear	Unclear
Mo and Zhao [25]	Unclear	Unclear	N	N	N	Unclear	Unclear
Song and Zhang [33]	Unclear	Unclear	N	Y	N	Unclear	Unclear
Zeng et al. [34]	Unclear	Unclear	N	N	N	Unclear	Unclear
Zeng [26]	Unclear	Unclear	N	N	N	Unclear	Unclear
Zhang [35]	Unclear	Unclear	N	Y	N	Unclear	Unclear

**Table 3:** Adverse events.

Symptom	Reported trails	Cases reported
Dry mouth	4 [10, 16, 17, 60]	14
Fullness of the head	4 [9–12]	10
Dryness heat	2 [10, 13]	7
Insomnia	1 [13]	3
Dysphoria	1 [14]	2
Skin itching	1 [15]	1
Tachycardia	1 [16]	1
Feverish dysphoria	2 [17, 18]	5
Flushing of face and tidal fever	1 [19]	8
Dizziness due to low blood pressure	1 [20]	1
Gastrointestinal discomfort	1 [20]	1
Palpitation	1 [18]	2

## References

[1] Haldeman GA, Croft JB, Giles WH, Rashidee A (1999). Hospitalization of patients with heart failure: national hospital discharge survey, 1985 to 1995. *American Heart Journal*.

[2] Gu D, Huang G, He J (2003). Investigation of prevalence and distributing feature of chronic heart failure in Chinese adult population. *Chinese Journal Cardiology*.

[3] Rosamond W, Flegal K, Furie K (2008). Heart disease and stroke statistics-2008 Update: a report from the American heart association statistics committee and stroke statistics subcommittee. *Circulation*.

[4] Swedberg K, Cleland J, Dargie H (2005). Guidelines for the diagnosis and treatment of chronic heart failure: executive summary (update 2005). *European Heart Journal*.

[5] Ramani GV, Uber PA, Mehra MR (2010). Chronic heart failure: contemporary diagnosis and management. *Mayo Clinic Proceedings*.

[6] Fu S, Zhang J, Menniti-Ippolito F (2011). Huangqi injection (a traditional chinese patent medicine) for chronic heart failure: a systematic review. *PLoS ONE*.

[7] China Pharmacopoeia Committee (2005). *Pharmacopoeia of the People's Republic of China*.

[8] Ji X-F, Yang L, Zhang M-Y, Li C-S, Wang S, Cong L-H (2011). Shen-Fu injection attenuates postresuscitation myocardial dysfunction in a porcine model of cardiac arrest. *Shock*.

[9] Li DQ, Zheng P, Lin X (2010). Curative effect of shenfu injection and furosemide on intractable heart failure. *Strait Pharmaceutical Journal*.

[10] Song SQ, Cheng HH, Huang PD (2006). Curative effect of shenfu injection on chronic contractive heart failure. *Journal of Sichuan of Traditional Chinese Medicine*.

[11] Zhao H, Dong ZL, Chen J (2004). Curative effect of shenfu injection on congestive heart failure. *Chinese Journal of Integrative Medicine on Cardio-/Cerebrovascular Disease*.

[12] Lei WG, Zhao HG, Fang YB (2003). Curative effect of shenfu injection on congestive heart failure. *Journal of Emergency in Traditional Chinese Medicine*.

[13] Huang HL (1999). The treatment of western medicine with Shenfu injection of 38 cases in elderly patients with acute left heart failure. *Chinese Journal of Integrated Traditional and Western Medicine*.

[14] Chen JH, Liu RJ (2007). Curative observation of metoprolol with shenfu injection for chronic congestive heart failure. *Internal Medicine of China*.

[15] Deng XY, Tang CS (2011). Observation of shengfu injection for chronic heart failure. *Medical Journal of West China*.

[16] Tian J, Gong Y (2003). The Clinical curative effect of congest heart failure (CHF) treated with shenfu injection. *Chinese Journal of Information on Traditional Chinese Medicine*.

[17] Hou XL, Hong JK (2004). Shenfu injection adjuvent therapy for chronic congestive heart failure. *Zhejiang Journal of Integrated Traditional Chinese and Western Medicine*.

[18] Liu J, Sun HL (2009). Clinical observation of shenfu injection for 28 cases of congestive heart failure. *Asia-Pacific Traditional Medicine*.

[19] Zhou JS (2004). Curative effect of western medicine combined with shenfu injection on chronic congestive heart failure. *Chinese Journal of Cardiovascular Rehabilitation Medicine*.

[20] Liu SM, Jin WJ, Zhu GY (2008). Clinical study of shenfu—injection combination enalapril and metoprolol on treating chronic congestive heart failure. *Chinese Archives of Traditional Chinese Medicine*.

[21] Kim S, Shin BC, Lee MS (2011). Red ginseng for type 2 diabetes mellitus: a systematic review of randomized controlled trials. *Chinese Journal of Integrative Medicine*.

[22] Guo Q, Fang BJ, Chen H (2009). Clinical observation of shenfu injection for heart failure of acute myocardial infarction. *Journal of Liaoning University of TCM*.

[23] Guo J, Lu JW, Wu XH (2008). Clinical observation of mechanical ventilation and shenfu injection for acute left heart failure (yang deficiency and water excess syndrome). *Journal of Guiyang College of Traditional Chinese Medicine*.

[24] Huang WQ, Tang RD, Lv WX (2008). Shengfu injection for treatment of acute left heart failure. *Journal of Integrative Medicine on Cardio/Cerebrovascular Disease*.

[25] Mo CR, Zhao KM (2002). Shenfu injection as adjuvant therapy in treating 36 patients with acute myocardial infarction with heart failure. *Chinese Journal of Integrated Traditional and Western Medicine*.

[26] Zeng YL (2005). Shenfu Injection as adjuvant therapy in treating 54 patients with Acute myocardial infarction with heart failure. *Chinese Practical Journal of Rural Doctor*.

[27] Geng XY, Lin XD, Wang YB (2006). Clinical study of shenfu injection for congestive heart failure. *Journal of Emergency in Traditional Chinese Medicine*.

[28] Wang WG (2010). Clinical observation of shenfu injection for chronic congestive heart failure. *Journal of Emergency in Traditional Chinese Medicine*.

[29] Zhang Y (2008). The treatment of conventional therapy with Shenfu Injection for 100 patients with Chronic Congestive Heart Failure. *Heilongjiang Medicine Journal*.

[30] Zhang WX, Pan G (2007). Curative effect of shenmai injection and shenfu injection on chronic congestive heart failure. *Modern Journal of Integrated Traditional Chinese and Western Medicine*.

[31] Zhou ZT, Li YW, Zhu HM (2005). Influence of shenfu injection on C-Reactive Protein in patients with heart failure caused by coronary heart diseases. *Tianjin Journal of Traditional Chinese Medicine*.

[32] Tu YP, Gong AB, Li NQ (2011). Study of shenfu injection on the treatment of congestive heart failure. *Hebei Journal of Traditional Chinese Medicine*.

[33] Song Q, Zhang XF (2001). Curative effect of shenfu injection on the treatment of 38 cases of acute myocardial infarction with heart failure. *Journal of Jining Medical college*.

[34] Zeng Y, Wu JB, Wang ZB (2009). Curative effect of shenfu injection on myocardial infarction with heart failure. *International Medicine & Health Guidance News*.

[35] Zhang HX (2011). Curative effect of shenfu injection on heart failure of acute myocardial infarction. *Chinese Journal of Ethnomedicine and Ethnopharmacy*.

[36] Han WF, Li EH (1999). Curative effect of shenfu injection on congestive heart failure. *Liaoning Journal of Traditional Chinese Medicine*.

[37] Ju YS (2009). Curative effect of shenfu injection on chronic heart failure. *China Practical Medicine*.

[38] Luo XY, Zhang FR, He RM (2009). Efficacy of shenfu injection as adjuvant therapy in treating patients of ischemic cardiomyopathy with heart insufficiency. *Chinese Journal of Integrated Traditional and Western Medicine*.

[39] Wang WM (2009). Clinical observation of shenfu injection for heart insufficiency of hypertension. *Journal of Emergency in Traditional Chinese Medicine*.

[40] Wu HY, Ye MS, Yi YX (2006). Treating effect of shenfu injection in elder patients with coronary heart disease. *Chinese Contemporary Medical Science*.

[41] Zi Y, Li ZY (2007). Clinical study of western medicine and Chinese medicine for chronic congestive heart failure. *Jiangsu Journal of Traditional Chinese Medicine*.

[42] Zhang H (2009). The experience of treating in 60 cases of chronic heart failure with shenfu injection. *Ningxia Medical Journal*.

[43] Zhang ZM (2003). Shenfu injection treatment of 30 cases in elderly patients with congestive heart failure of coronary heart disease. *Fujian Journal of Traditional Chinese Medicine*.

[44] Hong ML (2000). Curative effect of shenfu injection on congestive heart failure of chronic pulmonary heart disease. *Fujian Journal of Traditional Chinese Medicine*.

[45] Wu HJ, Duan SW (2009). Clinical study of shenfu injection for heart failure of coronary heart disease. *Chinese Journal of Integrative Medicine on Cardio-/ Cerebrovascular Disease*.

[46] Ru HG (2002). Curative effect of shenfu injection on congestive heart failure of chronic pulmonary heart disease. *Zhejiang Journal of Traditional Chinese Medicine*.

[47] Zhan LS, Yang CL (2008). Curative effect of metoprolol with shenfu injection for chronic congestive heart failure. *Chinese Journal of Rural Medicine*.

[48] Ma HW, Wu R, Hao RJ (2005). Clinical observation of shenfu injection for chronic contractive heart failure. *Chinese Journal of Integrative Medicine on Cardio-/ Cerebrovascular Disease*.

[49] Guo JJ, Guo YJ, Qiao JF (2006). Effect of shenfu injection on cardiac function and myocardial fibrosis of ischemic cardiomyopathy. *Journal of Fujian college of Traditional Chinese Medicine*.

[50] Liu SS, Chan PC (2007). Curative effect of shenfu injection as adjuvent therapy in treamting 50 patients with chronic congestive heart failure. *Shandong Medical Journal*.

[51] Chen XL, Li FX (2009). Curative effect of sodium nitroprusside with shenfu injection on intractable congestive heart failure. *Chinese Journal of Difficult and Complicated Cases*.

[52] Chen XB, Hou SR, Huang ZH (2009). Impact of shenfu injection on cardiac function and plasma NT—proBNP level in patients with congestive heart failure. *Journal of Emergency in Traditional Chinese Medicine*.

[53] Huang T (2009). Clinical study of shenfu injection for elderly chronic heart failure. *Journal of Emergency in Traditional Chinese Medicine*.

[54] Yang ZY, Dong JY, Miao LH (2010). Clinical observation of shenfu injection in elderly patients with chronic heart failure. *Journal of Emergency in Traditional Chinese Medicine*.

[55] Chen HY ( 2011). Impact of shenfu injection on central venous pressure and NT-pro brain natriuretic polypeptide in patients with chronic heart failure. *Fujian Journal of TCM*.

[56] Chen ZG, Li HJ, Zhang SR (2009). Evaluation of shenfu injection in patients with dilated cardiomyopathy. *Medical Innovation of China*.

[57] Lv G (2010). Curative effect analysis of shenfu injection for 31 cases of dilated cardiomyopathy. *China Practical Medicine*.

[58] Tan LJ, Chu KQ, An Y (2010). Effect of shenfu injection on cardiac function and brain natriuretic polypeptide in patients with heart failure. *Journal of Emergency in Traditional Chinese Medicine*.

[59] Zhou G (2010). Curative effect of shenfu injection on intractable heart failure. *Journal of Qiqihar Medical College*.

[60] Fan SM (2010). Clinical observation of shenfu injection on treating congestive heart failure. *Journal of Medical Forum*.

[61] Bao GH, Yu LH (2011). Treating 30 cases of chronic congestive heart failure with shenfu injection. *Chinese Medicine Modern Distance Education of China*.

[62] Jiang QY (2007). Curative effect of shenfu injection on chronic congestive heart failure. *Guide of China Medicine*.

[63] Tu QY, Yang YL (2009). Clinical observation on treating heart failure and atrial fibrillation with slow ventricular rate with shen—fu injection. *Liaoning Journal of Traditional Chinese Medicine*.

[64] Wu YB, Wang CJ (2010). Curative effect of shenfu injection on elderly congestive heart failure. *Chinese Journal of Convalescent Medicine*.

[65] Yao J, Lu XR (2010). Clinical observation of shenfu injection for chronic congestive heart failure. *The Journal of Medical Theory and Practice*.

[66] Yu JY (2011). Impact of shenfu injection on NT-pro brain natriuretic polypeptide leveL and cardiac functionl in patients with chronic heart failure. *Modern Medicine & Health*.

[67] Di ST (2010). Clinical study of shenfu injection for heart failure. *Shanxi Medical Journal*.

[68] Gao ZW, Liu YX, Lv ZM (1999). Observation of combining with shengfu injection for congestive heart failure. *Medical Journal of Communications*.

[69] Gu XM, Yin J, Wang QZ (2005). Analysis of the effect of shenfu injection on cardiac function in 55 Patients with heart failure. *Chinese Journal of Integrative Medicine on Cardio-/ Cerebrovascular Disease*.

[70] He XJ (2010). Curative effect of shenfu injection on 120 cases of chronic heart insufficiency. *Journal of China Traditional Chinese Medicine Information*.

[71] Jia Q, Yang X (2005). Observation and nursing of shengfu injection for chronic heart failure. *Today Nurse*.

[72] Li H, Liu X, Huang NB (2002). Curative effect of shenfu injection on congestive heart failure. *Journal of Emergency in Traditional Chinese Medicine*.

[73] Li QH, Liu X, Yang TL (2009). Clinical observation of large dose of shenfu injection for intensive intractable heart failure. *Modern Medicine Journal of China*.

[74] Liu XJ (2009). The changes of plasma urotensin II and adrenomedullin in chronic deart failure and the intervention studies with Shenfu injection. *Medical Journal of West China*.

[75] Liu Y (2008). Clinical observation of 40 cases of shenfu injection on chronic congestive heart failure. *Journal of Emergency in Traditional Chinese Medicine*.

[76] Luo SP, Li G, Zhang Y (2008). The treatment of sodium nitroprusside with shenfu injection on chronic congestive heart failure. *Chinese Journal of Integrative Medicine on Cardio-/Cerebrovascular Disease*.

[77] Ma SB (2011). Shenfu injection treatment of 50 cases of congestive heart failure. *Journal of Practical Traditional Chinese Internal Medicine*.

[78] Shang Y (2011). Treating 120 cases of chronic heart failure with shenfu injection. *Chinese and Foreign Medical Research*.

[79] Zhang L (2011). Curative effect of shenfu injection on chronic heart failure. *Medical Journal of West China*.

[80] Tian LN (2010). Impact of shenfu injection on cardiac function index and clinical effect in patients with heart failure of coronary heart disease. *Shanxi Medical Journal*.

[81] Wang YY, Qiao ZL, Yang JX (2011). Study of shenfu injection on the treatment of 40 cases of congestive heart failure with slow arrhythmia. *Journal of Emergency in Traditional Chinese Medicine*.

[82] Yang Y, Wu XH (2009). Clinical observation of shenfu injection for 30 cases of dilated cardiomyopathy with Heart Failure. *Yunnan Journal of Traditional Chinese Medicine and Materia Medica*.

[83] Yin H (2008). Clinical observation of shenfu injection in elderly patients with intractable congestive heart failure. *Journal of Medical Forum*.

[84] Yu GY, Qu HJ, Song JM (2005). Curative effect of shenfu injection on congestive heart failure. *Chinese Journal of Integrative Medicine on Cardio-/Cerebrovascular Disease*.

[85] Zhang AP, Song GP, Cai JS (2011). Study of shenfu injection on the treatment of 40 cases of chronic congestive heart failure. *Journal of Emergency in Traditional Chinese Medicine*.

[86] Cui ZJ (2000). Curative effect of shenfu injection on chronic congestive heart failure. *Modern Journal of Integrated Traditional Chinese and Western Medicine*.

[87] Wang XM, Ye XL (2001). The clinical curative effect of shenfu injection on congestive heart failure. *Chinese Synthetical Medicine*.

[88] Jian YP, Chen YM (2002). Shenfu injection treatment of 64 cases of intractable congestive heart failure. *Fujian Journal of Traditional Chinese Medicine*.

[89] Pan MJ, Yue RS, Liang C (2003). Curative effect of shenfu injection on congestive heart failure. *Journal of Chengdu University of Traditional Chinese Medicine*.

[90] Su YS (2003). Shenfu injection treatment of 32 cases for heart failure with arrhythmia. *Journal of Emergency in Traditional Chinese Medicine*.

[91] Zhao XX (2011). Clinical curative effect of shenfu injection on chronic heart failure. *Journal of Qiqihar University of Medicine*.

[92] Lei HL, Li JM (2004). The treatment of shenfu injection for congestive heart failure. *Chinese Journal of Integrative Medicine on Cardio-/Cerebrovascular Disease*.

[93] Li ZH (2004). Clinical analysis of treamting heart insufficiency of chronic pulmonary heart disease with shenfu injection. *Journal of Chinese Medicine Research*.

[94] Liu YJ, Liu XY, Li F (2005). The clinical curative effect of shenfu injection on heart failure. *Practical Pharmacy And Clinical Remedies*.

[95] Jin XP, Guo JS (2007). The effect of Shenfu injection on endothelium function of patients with congestive heart failure. *Journal of Emergency in Traditional Chinese Medicine*.

[96] Li LZ (2007). Treating 48 cases of congestive heart failure of chronic pulmonary heart disease with shenfu injection. *Henan Traditional Chinese Medicine*.

[97] Dou J (2008). Clinical observation of shenfu injection as adjuvent therapy in treating 41 patients with congestive heart failure. *Forum on Traditional Chinese Medicine*.

[98] He HX (2008). The treatment of 35 cases of sodium nitroprusside with shenfu injection on chronic congestive heart failure. *Guangming Journal of Chinese Medicine*.

[99] Ma JJ, Huang XL (2008). Curative effect of shenfu injection on chronic congestive heart failure. *Chinese Community Doctors*.

[100] Wang Q (2008). Curative effect of shenfu injection on acute exacerbation of pulmonary heart disease with congestive heart failure. *Journal of Emergency in Traditional Chinese Medicine*.

[101] Fan DB, Qin XP, Bai HH (2009). Clinical observation of shenfu injection for 62 patients with congestive heart failure of pulmonary heart disease. *Journal of Emergency in Traditional Chinese Medicine*.

[102] Guo YF, Li XB, Song K (2009). Curative effect of Chinese medicine integrated with western medicine on 128 cases of acute heart failure and respiratory failure. *Journal of Emergency in Traditional Chinese Medicine*.

[103] Qiu WW (2010). Curative effect of shenfu injection on chronic heart failure. *Journal of Emergency in Traditional Chinese Medicine*.

[104] Wang GL, Wang J (2010). Treating 50 cases of chronic heart failure with shenfu injection. *Chinese Journal of the Practical Chinese with Modern Medicine*.

[105] Zhu CZ, Ma Y (2010). Curative Effect of shenfuinjection on chronic heart failure. *Journal of Emergency in Traditional Chinese Medicine*.

[106] Song JJ (2011). Curative effect of shenfu injection on silicosis with chronic pulmonary heart disease and heart failure. *Hebei Medical Journal*.

[107] Guyton AC (2006). *Textbook of Medical Physiology*.

[108] Mohamed AL, Yong J, Masiyati J, Lim L, Tee SC (2004). The prevalence of diastolic dysfunction in patients with hypertension referred for echocardiographic assessment of left ventricular function. *Malaysian Journal of Medical Sciences*.

[109] Bhalla V, Willis S, Maisel AS (2004). B-type natriuretic peptide: the level and the drug–partners in the diagnosis of congestive heart failure. *Congestive Heart Failure*.

[110] Sze FKH, Yeung FF, Wong E, Lau J (2005). Does Danshen improve disability after acute ischaemic stroke?. *Acta Neurologica Scandinavica*.

[111] Cao H, Liu J, Lewith GT (2010). Traditional Chinese medicine for treatment of fibromyalgia: a systematic review of randomized controlled trials. *Journal of Alternative and Complementary Medicine*.

[112] Huo YZ, Mao JY, Wang XL (2011). SFI for patients with heart failure: a systematic review. *Chinese Journal of Evidence-based Medicine*.

[113] Bin XF (2010). Systematic Review and meta-analysis of Shenfu injection for congestive heart failurev. *Journal of Guiyang College of Traditional Chinese Medicine*.

[114] The European Agency for the Evaluation of Medicinal Products (2007). Note for guidance on clinical investigation of medicinal products for the treatment of cardiac failure. *TGA Internet Site, CPMP/EWP/235/95 Rev*.

[115] Zhang YQ, Han M, Liu ZJ (2012). Chinese herbal formula xiao yao san for treatment of depression: a systematic review of randomized controlled trials. *Evidence-Based Complementary and Alternative Medicine*.

[116] Liu J, Manheimer E, Shi Y, Gluud C (2004). Chinese herbal medicine for severe acute respiratory syndrome: a systematic review and meta-analysis. *Journal of Alternative and Complementary Medicine*.

[117] Wu T, Li Y, Bian Z, Liu G, Moher D (2009). Randomized trials published in some Chinese journals: how many are randomized?. *Trials*.

[118] Jin Z, Yu D, Zhang L (2010). A retrospective survey of research design and statistical analyses in selected chinese medical journals in 1998 and 2008. *PLoS ONE*.

