# Supplementary material for: Chinese Medicine Shenfu Injection for Heart Failure: A Systematic Review and Meta-Analysis
Source: Evid Based Complement Alternat Med. 2012 Apr 24;2012:713149. doi: 10.1155/2012/713149 (PMC3348640; doi:10.1155/2012/713149)
Supplement: Supplementary file 1 — Compared with routine treatment and/or device support, SFI combined with routine treatment and/or device support showed better effect on ultrasonic cardiography. Significance are showed in most parameters of cardiography, namely, LVEF (WMD: 6.31; 95% CI [5.18, 7.44], P<0.01), SV (WMD:7.25; 95%CI [4.60, 9.90], P<0.01), CO (WMD: 0.67; 95%CI [0.47, 0.87], P<0.01), CI (WMD: 0.36; 95%CI [0.23, 0.48], P<0.01) and E/A ratio (WMD:0.15; 95%CI [0.08, 0.22], P<0.01). There were no significant difference in LVDd (WMD: −1.59; 95% CI [-5.29, 2.12], P=0.40), systolic blood pressure (WMD: −0.07; 95%CI [−0.42, 0.27], P=0.68) and diastolic blood pressure (WMD: −0.37; 95% CI [−0.97, 0.23], P=0.22) between SFI and routine treatment groups. [file 713149.f1.doc]

Supplement Figure 1 Forest plot of comparison: heart rate.

Supplement Figure 2 Forest plot of comparison: SBP.

Supplement Figure 3 Forest plot of comparison: DBP.

Supplement Figure 4 Forest plot of comparison: LVEF.

Supplement Figure 5 Forest plot of comparison: SV.

Supplement Figure 6 Forest plot of comparison: CO.

Supplement Figure 7 Forest plot of comparison: CI.

Supplement Figure 8 Forest plot of comparison: E/A.

Supplement Figure 9 Forest plot of comparison: LVDd.
